# Supplementary material for: Association Between Intensity of Physical Activity in Pregnancy and Gestational Diabetes in a Multi-Ethnic Population: Results from the PROMOTE Cohort Study
Source: Nutrients. 2025 Nov 7;17(22):3500. doi: 10.3390/nu17223500 (PMC12655390; doi:10.3390/nu17223500)
Supplement: Supplementary file 1 [file nutrients-17-03500-s001.zip › S1_SociodemographicQuestionnaire.pdf.pdf.pdf]

Study Code/ID: ___________________________

Date: ___________________________

Sociodemographic Questionnaire

1. How many people live in your household (including yourself)?
   - [ ] Number of people is ___
   - [ ] Don't know
   - [ ] Prefer not to say
2. How many of the people living in the household are children 0-15 years of age?
   - [ ] Number of children living in the household is ___
   - [ ] Don't know
   - [ ] Prefer not to say
3. What is the level of the highest qualification you have completed?
   - [ ] Completed primary school
   - [ ] Completed years 7-9
   - [ ] Completed School Certificate/Intermediate/Year 10/4th Form
   - [ ] Completed HSC/Leaving/Year 12/6th Form
   - [ ] TAFE Certificate or Diploma
   - [ ] University, CAE or some other tertiary institute degree or higher
   - [ ] Other [specify]
   - [ ] Don't know
   - [ ] Prefer not to say

4. In the last week, which of the following best describes your employment status?

[ ] Wage earner (or salaried)

[ ] Self-employed

[ ] Employed but on leave (e.g. maternity leave)

[ ] Unpaid work in a family business

[ ] Unpaid work

[ ] Did not have a job

[ ] Don’t know/not sure

[ ] Prefer not to say

5. Which of the following ranges best describes your <u>household's</u> gross income?

[ ] Less than $25,000 per annum

[ ] $25,000 to less than $50,000

[ ] $50,000 to less than $75,000

[ ] $75,000 to less than $100,000

[ ] $100,000 to less than $150,000

[ ] $150,000 to less than $200,000

[ ] $200,000 or more

[ ] Don't know

[ ] Prefer not to say
