# Supplementary material for: Association Between Intensity of Physical Activity in Pregnancy and Gestational Diabetes in a Multi-Ethnic Population: Results from the PROMOTE Cohort Study
Source: Nutrients. 2025 Nov 7;17(22):3500. doi: 10.3390/nu17223500 (PMC12655390; doi:10.3390/nu17223500)
Supplement: Supplementary file 1 [file nutrients-17-03500-s001.zip › S2_ActiveAustraliaSurvey.pdf]

Study Code/ID: ____________________

Date: ____________________

Active Australia Survey

The next questions are about any physical activities that you may have done in the last week:

1. In the last week, how many times have you walked continuously, for at least 10 minutes, for recreation, exercise or to get to or from places?

   [ ][ ] times

2. What do you estimate was the total time that you spent walking in this way in the last week?

   In hours and/or minutes

   [ ][ ][ ] minutes

   [ ][ ] hours

3. In the last week, how many times did you do any vigorous gardening or heavy work around the yard, which made you breathe harder or puff and pant?

   [ ][ ] times

4. What do you estimate was the total time that you spent doing vigorous gardening or heavy work around the yard in the last week?

In hours and/or minutes

minutes

hours

The next questions exclude household chores, gardening or yardwork:

5. In the last week, how many times did you do any vigorous physical activity which made you breathe harder or puff and pant? (e.g. jogging, cycling, aerobics, competitive tennis)

times

6. What do you estimate was the total time that you spent doing this vigorous physical activity in the last week?

In hours and/or minutes

minutes

hours

7. In the last week, how many times did you do any other more moderate physical activities that you have not already mentioned? (e.g. gentle swimming, social tennis, golf)

times

8. What do you estimate was the total time that you spent doing these activities in the last week?

In hours and/or minutes

			minutes

		hours

To what extent do you agree or disagree with the following statements about physical activity and health?

9(a) Taking the stairs at work or generally being more active for at least 30 minutes each day is enough to improve your health.

strongly disagree	disagree	neither agree nor disagree	agree	strongly agree

9(b) Half an hour of brisk walking on most days is enough to improve your health.

strongly disagree	disagree	neither agree nor disagree	agree	strongly agree

9(c) To improve your health it is essential for you to do vigorous exercise for at least 20 minutes each time, three times a week.

strongly disagree	disagree	neither agree nor disagree	agree	strongly agree

9(d) Exercise doesn't have to be done all at one time—blocks of 10 minutes are okay.

strongly disagree	disagree	neither agree nor disagree	agree	strongly agree

9(e) Moderate exercise that increases your heart rate slightly can improve your health.

strongly disagree	disagree	neither agree nor disagree	agree	strongly agree
