# Supplementary material for: Association Between Intensity of Physical Activity in Pregnancy and Gestational Diabetes in a Multi-Ethnic Population: Results from the PROMOTE Cohort Study
Source: Nutrients. 2025 Nov 7;17(22):3500. doi: 10.3390/nu17223500 (PMC12655390; doi:10.3390/nu17223500)
Supplement: Supplementary file 1 [file nutrients-17-03500-s001.zip › nutrients-3956194-supplementary material.pdf]

Supplementary Materials

Supplementary Table S1 - Socio-Economic Indexes for Areas Scores

			N = 351
	SEIFA—socio-economic disadvantage, n (%)		
		Quintile 1	90 (26)
		Quintile 2	41 (12)
		Quintile 3	97 (28)
		Quintile 4	23 (7)
		Quintile 5	100 (28)
	SEIFA—economic resources, n (%)		
		Quintile 1	182 (52)
		Quintile 2	25 (7)
		Quintile 3	39 (11)
		Quintile 4	4 (1)
		Quintile 5	101 (29)
	SEIFA—education and occupation, n (%)		
		Quintile 1	4 (1)
		Quintile 2	31 (9)
		Quintile 3	67 (19)
		Quintile 4	27 (8)
		Quintile 5	222 (63)

Supplementary Table S2. Characteristics associated with any moderate or vigorous physical activity

		ANY MODERATE/VIGOROUS (N = 116; 28%)	NO MODERATE/VIGOROUS (N = 300; 72%)	P
BASELINE CHARACTERISTICS	Maternal Age	32 (28–35)	33 (30–36)	0.178
	BMI	25 (22–31)	25 (22–29)	0.984
	Any Children in Household			
	Yes	73% (97)	68% (234)	0.259
	No	27% (35)	32% (112)	
EDUCATIONAL ATTAINMENT	Incomplete School	2% (3)	2% (10)	0.323
	Complete School	9% (12)	9% (30)	
	School + TAFE	24% (31)	17% (57)	
	School + Uni	65% (84)	72% (246)	
FINANCIAL STATUS	Financial Autonomy			
	Yes	77% (103)	69% (241)	0.165
	No	19% (25)	27% (94)	
	Unknown	4% (5)	4% (15)	
	Household Income			

	<50K	6% (8)	5% (18)	0.108
	50-100K	19% (25)	22% (78)	
	100-200K	38% (51)	32% (111)	
	>200K	20% (27)	14% (50)	
	Unknown	17% (22)	26% (90)	
**MENTAL HEALTH SCREENING**	DASS-Anxiety			
**DASS**	Normal	78% (64)	74% (207)	0.631
	Mild	9% (7)	8% (22)	
	Moderate+	13% (11)	18% (50)	
	DASS-Depression			
	Normal	91% (75)	90% (253)	0.862
	Mild	5% (4)	6% (18)	
	Moderate+	4% (3)	3% (9)	
	DASS-Stress			
	Normal	70% (57)	66% (183)	0.788
	Mild	6% (5)	8% (21)	
	Moderate+	24% (20)	27% (75)	
**EPDS**	Low	87% (116)	89% (311)	0.658
	Moderate	9% (12)	7% (23)	
	High	4% (5)	4% (14)	
**ETHNICITY**	Middle Eastern	21% (28)	16% (58)	0.008
	Other	11% (15)	8% (29)	
	S Asian	24% (32)	41% (144)	
	SE Asian	17% (23)	17% (61)	
	White	26% (35)	17% (60)	
**MEDICAL AND OBSTETRIC HISTORY**	Multiparous			
	Yes	73% (97)	68% (240)	0.367
	No	27% (36)	32% (112)	
	Hx GDM			
	Yes	15% (20)	14% (50)	0.930
	No	85% (113)	86% (302)	
	Hx PCOS			
	Yes	10% (13)	8% (29)	0.722
	No	90% (120)	92% (323)	
	Hx Recurrent Miscarriage			
	Yes	17% (22)	12% (44)	0.313
	No	83% (111)	88% (308)	
	Assisted Conception			
	Yes	5% (6)	6% (21)	0.688
	No	95% (127)	94% (331)	
**BREASTFEEDING HISTORY**	High Intensity BLISS			

	Yes	84% (76)	87% (194)	0.470
	No	16% (15)	13% (28)	

Supplementary Table S3. Characteristics of those with GDM vs those without

		GDM (N = 104; 25%)	NO GDM (N = 312; 75%)	P
**BASELINE CHARACTERISTICS**	Maternal Age	34 (31–37)	32 (29–35)	0.001
	BMI	26.7 (23.6–31.3)	24.5 (21.8–28.0)	<0.001
	Any Children in Household			
	Yes	78 (76%)	211 (69%)	0.238
	No	25 (24%)	95 (31%)	
**EDUCATIONAL ATTAINMENT**	Incomplete School	3 (3%)	7 (2%)	0.762
	Complete School	7 (7%)	29 (10%)	
	School + TAFE	21 (21%)	53 (17%)	
	School + Uni	71 (70%)	214 (71%)	
**FINANCIAL STATUS**	Financial Autonomy			
	Yes	68 (66%)	229 (74%)	0.099
	No	27 (26%)	72 (23%)	
	Unknown	8 (8%)	10 (3%)	
	Household Income			
	<50K	6 (6%)	16 (5%)	0.869
	50-100K	21 (20%)	64 (21%)	
	100-200K	33 (32%)	106 (34%)	
	>200K	14 (14%)	50 (16%)	
	Unknown	29 (28%)	72 (23%)	
**MENTAL HEALTH SCREENING**	DASS-Anxiety			
**DASS**	Normal	62 (79%)	167 (76%)	0.336
	Mild	8 (10%)	17 (8%)	
	Moderate+	8 (10%)	37 (17%)	
	DASS-Depression			
	Normal	71 (91%)	205 (92%)	0.039
	Mild	7 (9%)	8 (4%)	
	Moderate+	0 (0%)	9 (4%)	
	DASS-Stress			
	Normal	52 (67%)	153 (69%)	0.694
	Mild	5 (6%)	18 (8%)	
	Moderate+	21 (27%)	50 (23%)	
**MENTAL HEALTH SCREENING**				
**EPDS**	Low	90 (88%)	281 (91%)	0.217
	Moderate	10 (10%)	17 (5%)	

	High	2 (2%)	12 (4%)	
**ETHNICITY**	Middle Eastern	14 (13%)	65 (21%)	0.154
	Other	6 (6%)	30 (10%)	
	S Asian	47 (45%)	106 (34%)	
	SE Asian	19 (18%)	50 (16%)	
	White	18 (17%)	61 (20%)	
**MEDICAL AND OBSTETRIC HISTORY**	Multiparous			
	Yes	81 (78%)	215 (69%)	0.104
	No	23 (22%)	97 (31%)	
	Hx GDM			
	Yes	37 (36%)	17 (5%)	<0.001
	No	67 (64%)	295 (95%)	
	Hx PCOS			
	Yes	9 (%)	26 (8%)	1.000
	No	95 (91%)	296 (92%)	
	Hx Recurrent Miscarriage			
	Yes	20 (19%)	40 (13%)	0.147
	No	84 (81%)	272 (87%)	
	Assisted Conception			
	Yes	5 (5%)	16 (5%)	1.000
	No	99 (95%)	296 (95%)	
**BREASTFEEDING HISTORY**	High Intensity BLISS			
	Yes	62 (86%)	175 (87%)	0.998
	No	10 (14%)	26 (13%)	

Supplementary Table S4. Messaging agreement by physical activity

EXERCISE TYPE	MESSAGE 1	MESSAGE 2	MESSAGE 3	MESSAGE 4	MESSAGE 5
**PA GROUPS**	0.661	0.195	0.021	0.430	0.681
**INACTIVE**	79% (59)	87% (65)	61% (46)	67% (50)	83% (62)
**LOW ACTIVE**	82% (178)	89% (195)	71% (155)	73% (160)	86% (188)
**SUFFICIENTLY ACTIVE**	78% (150)	83% (160)	58% (112)	69% (132)	84% (162)
**ANY WALKING**	0.593	0.207	1.00	0.432	1.000
**YES**	79% (341)	86% (369)	64% (277)	71% (306)	85% (365)
**NO**	84% (46)	93% (51)	65% (36)	65% (36)	84% (47)
**ANY MODERATE**	1.00	1.00	0.261	0.603	1.00
**YES**	80% (68)	87% (74)	59% (50)	68% (58)	85% (72)
**NO**	80% (319)	86% (346)	66% (263)	71% (284)	85% (340)
**ANY VIGOROUS**	0.156	1.00	0.034	0.490	0.596
**YES**	86% (64)	86% (64)	76% (56)	74% (55)	88% (65)

**NO**	79% (323)	87% (356)	63% (257)	70% (287)	84% (347)
**ANY MODERATE OR VIGOROUS**	0.163	0.656	1.00	0.657	1.00
**YES**	84% (112)	88% (117)	65% (86)	72% (96)	85% (113)
**NO**	78% (275)	86% (303)	64% (227)	70% (246)	85% (299)

Message 1: Taking the stairs at work or generally being more active for at least 30 min each day is enough to improve your health.
Message 2: Half an hour of brisk walking on most days is enough to improve your health.

Message 3: To improve your health it is essential for you to do vigorous exercise for at least 20 minutes each time, three times a week.

Message 4: Exercise doesn't have to be done all at one time - blocks of 10 minutes are okay.

Message 5: Moderate exercise that increases your heart rate slightly can improve your health.
