# Supplementary figures and images for: Association Between Intensity of Physical Activity in Pregnancy and Gestational Diabetes in a Multi-Ethnic Population: Results from the PROMOTE Cohort Study
Source: Nutrients. 2025 Nov 7;17(22):3500. doi: 10.3390/nu17223500 (PMC12655390; doi:10.3390/nu17223500)

Supplementary Figure S1. Visual Summary of Key Findings

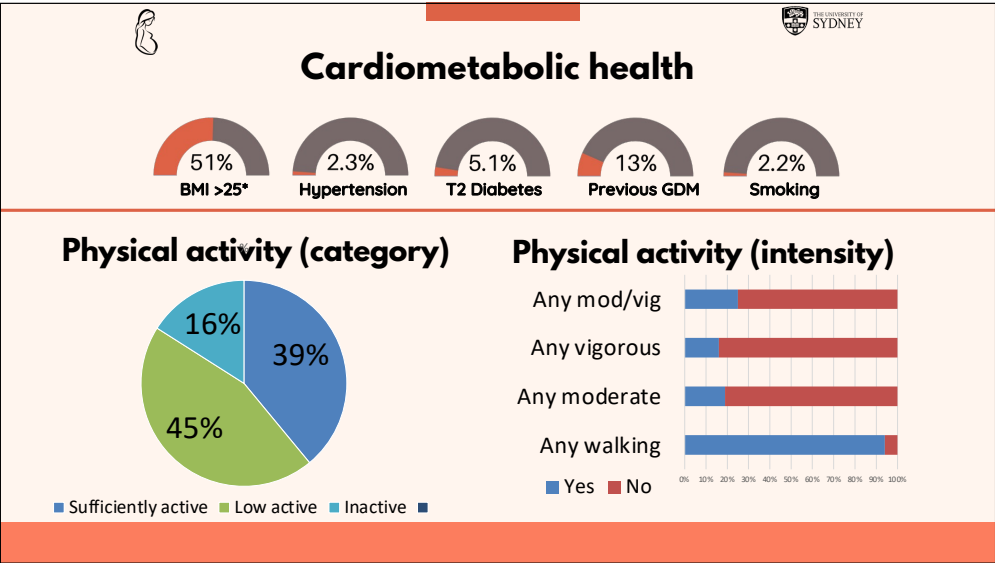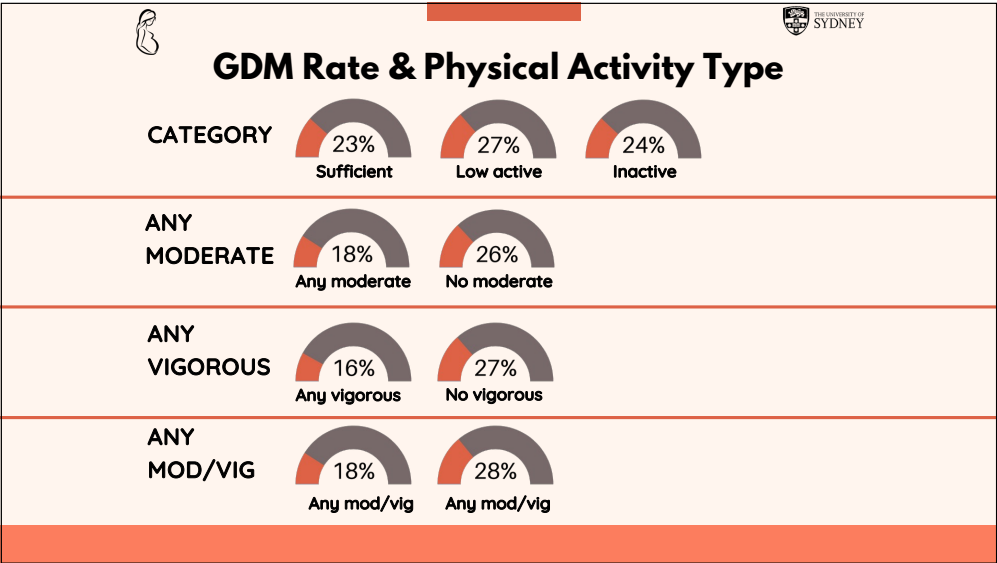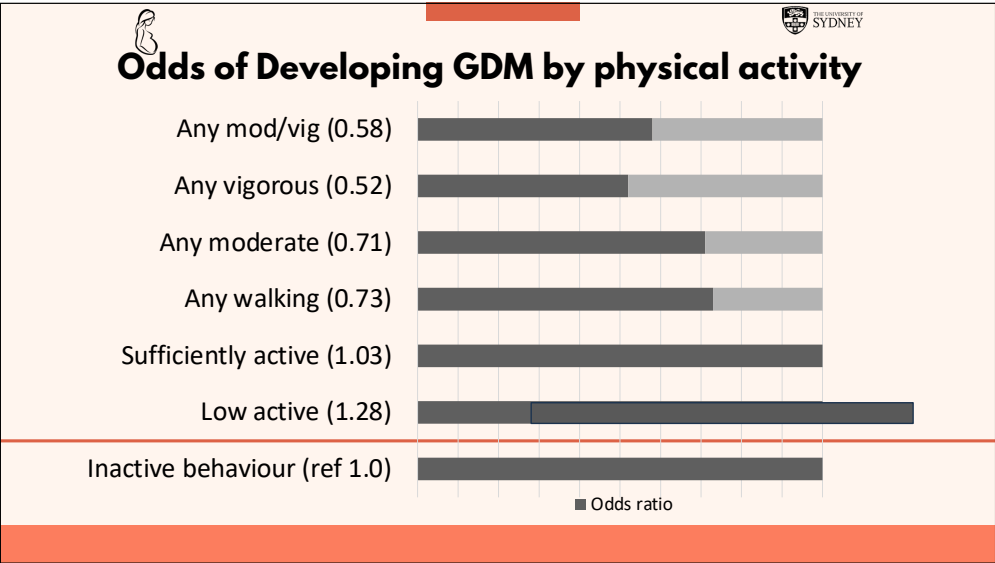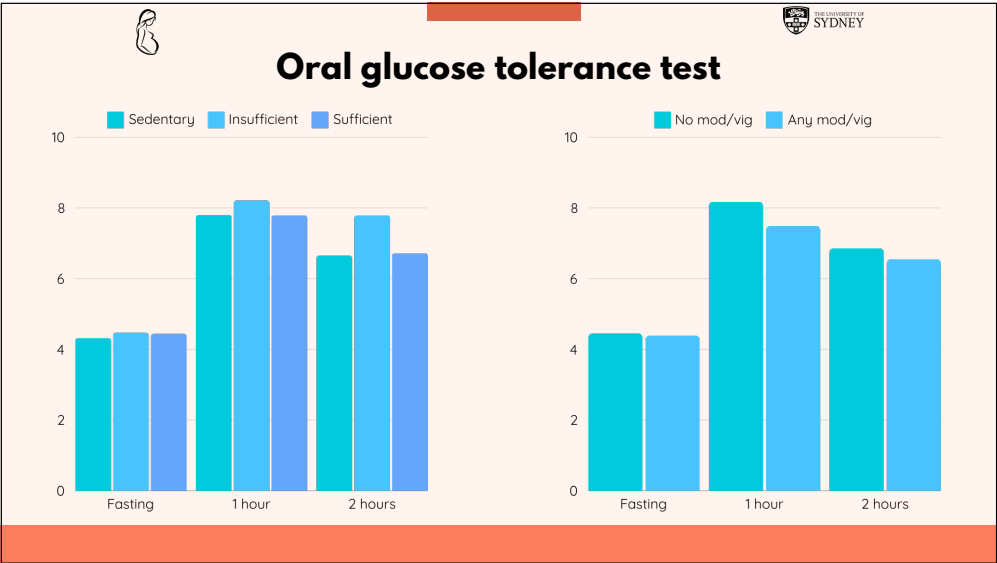

Supplement: Supplementary file 1 [file nutrients-17-03500-s001.zip › nutrients-3956194-figure s1.pdf]
